# Percutaneous Occlusion of Left Atrial Appendage: Growing Clinical Experience and Lack of Multicenter Randomized Clinical Trials

**DOI:** 10.5935/abc.20190210

**Published:** 2019-10

**Authors:** Cristiano F. Pisani, Mauricio Scanavacca

**Affiliations:** Unidade Clínica de Arritmia do Instituto do Coração (InCor) do Hospital das Clínicas da Faculdade de Medicina da Universidade de São Paulo (HC-FMUSP), São Paulo, SP - Brazil

**Keywords:** Atrial Fibrillation/therapy, Atrial Appendage/diagnotic, imaging, Prostheses and Implants/adverse effects, Stroke/prevention and control.

Left atrial appendage (LAA) closure as a prophylactic strategy for thromboembolic events in patients with atrial fibrillation (AF) has been performed for decades; initially during mitral valve repair surgeries^1^ and, more recently, in nonvalvular AF patients at high risk of embolism who do not tolerate the use of oral anticoagulants (OACs).

The idea of LAA occlusion as an alternative to chronic warfarin use emerged from observations of anatomopathological studies and during cardiac surgery that disclosed the LAA as the main site of thrombus formation in patients with nonvalvular atrial fibrillation.^2,3^

The evolution of cardiac access interventionist techniques, together with the development of specific prostheses for LAA occlusion, allowed the appendage closure to be performed percutaneously, using a minimally invasive procedure, making it simpler and not restricted to patients who would have undergone heart surgery.

The first prosthesis developed for this purpose, called PLAATO, was tested early in the last decade by Horst Sievert et al.^4^ and consisted of a nitinol structure, covered by an expanded polytetrafluoroethylene (ePTFE) occlusive membrane. The clinical study published in 2002 showed that the concept of percutaneous LAA occlusion was feasible; however, the prosthesis use was discontinued in 2005, due to the considerable number of complications such as cardiac tamponade, residual leaks, prosthesis protrusion towards the atrial cavity and, in some cases, lack of neo-endothelization of the prosthesis with formation of local thrombi.^5^ On the other hand, the experience obtained with the implantation of this prosthesis was important for the development of more effective devices.

Currently, two prostheses with different profiles are being used in clinical practice: the Watchman prosthesis sold by Boston Scientific and the Amplatzer Amulet device (evolution of the Amplatzer Cardiac Plug) sold by ABBOTT. Of these, only the Watchman prosthesis has been evaluated in two prospective, multicenter, and randomized clinical trials. The PROTECT-AF study (Watchman Left Atrial Appendage Closure Technology for Embolic Protection in Patients With Atrial Fibrillation),^6^ evaluated the effectiveness and safety of percutaneous LAA occlusion with the Watchman prosthesis, compared with oral anticoagulation with warfarin in 707 patients (463 in the intervention group) with nonvalvular AF and CHADS2 ≥ 1. The LAA occlusion (3 events per patient-year) met the noninferiority criterion compared to warfarin (4.9 events per patient-year) in the efficacy criterion; however, the LAA occluder implantation was associated with a higher number of adverse events, especially the occurrence of hemopericardium (4.8%), which was related to the interventionist’s learning curve in the prosthesis placement.

Due to safety concerns, the study was repeated (Watchman LAA Closure Device in Patients With Atrial Fibrillation Versus Long Term Warfarin Therapy - PREVAIL trial)^7^ with the same characteristics as the previous one, except for the greater experience of the operators. A total of 407 patients (269 in the intervention group) were included and at 18 months of follow-up, efficacy event rates (stroke, systemic embolization, and cardiovascular or unexplained death) of 0.064 were observed in the intervention group and 0.063 in the warfarin group, thus not meeting the pre-specified non-inferiority criteria previously obtained in the PROTEC-AF study, due to the very low number of events in the control group, a fact not observed in the previous and subsequent studies using warfarin.

However, the noninferiority criterion was met in the analysis of the second primary efficacy endpoint related to the event rate after 7 days of randomization. Also positive was the lower rate of prosthesis implant complications compared with the PROTECT AF study.

A complicating factor for the clinical implementation of the LAA occlusion strategy was the emergence of four new direct-acting oral anticoagulants (DOACs), supported by potent clinical studies showing no inferiority or even superiority of these new drugs over warfarin in patients with nonvalvular AF.^8,9^ Due to the practical use of DOACs, the indication of appendage occlusion devices has been postponed and considered only in patients who are intolerant to oral anticoagulants, or in those who experienced embolic events while using these drugs, although the effectiveness of the device has not been studied in randomized controlled trials.

Therefore, due to this heterogeneity of indications and the lack of randomized controlled trials, the records have become important. Reddy et al.^10^ evaluated 3822 consecutive cases of LAA occluder implantation based on Medicare data, showing a cardiac tamponade rate of 1.02%, most of them adequately treated with pericardiocentesis; however, the tamponade resulted in death in three cases. These rates were lower than those observed in clinical studies, although most devices were implanted by less experienced operators. Another European registry (EWOLUTION)^11^ also demonstrated a low complication rate, showing 34 (3.3%) adverse events among 1021 patients included in the study.

Two Brazilian registries have been recently published and suggested the safety of appendage occlusion device implantation. Guerios et al.,^12^ performed a multicenter registry and evaluated the results of 91 patients with nonvalvular AF (62% ineligible for anticoagulation) and high risk of stroke (CHA2DS2VASc 4.5 ± 1.5), submitted to the implantation of 96 prostheses, with the ACP (Amplatzer Cardiac Plug) being implanted in 94.6%. The implant success rate was 97.8%, with 7.2% of complications, with five pericardial effusions requiring pericardiocentesis, one non-dedicated device embolization and one gas embolism without sequelae. In this series, during a median follow-up of 346 days (128.6 patient-years), three non-procedure-related deaths were observed, as well as five cases of peri-prosthesis leakage, with thrombus formation next to the prosthesis in two, resolved with the return of anticoagulation and only two patients had stroke at the follow-up.

In the second registry, Marcio Costa et al.,^13^ evaluated 15 patients with nonvalvular AF and high risk of bleeding, submitted to implantation of the ACP prosthesis. In this small series, the procedure was successfully performed in all cases with no reports of hemopericardium or prosthesis displacement.

In this issue of the Brazilian Archives of Cardiology, Şahiner et al.^14^ disclose retrospective data from a center in Turkey, which included 60 patients submitted to implantation of the Amplatzer Amulet device. The main indication for the procedure was the occurrence of bleeding (usually gastrointestinal) in the presence of oral anticoagulation. The authors demonstrated that the implantation procedure was successful and safe in most patients. One patient had pulmonary artery rupture due to a probable direct injury by the prosthesis struts. In most patients, antiplatelet therapy consisted of ASA (100 mg) and clopidogrel (75 mg) for 6 months after the procedure, being maintained on single therapy after transesophageal echocardiography demonstrated the absence of periprosthetic leaks or thrombi. During a mean follow-up of 21 ± 15 months, none of the patients had a stroke but two patients had clinical symptoms of transient ischemic attack.

Thus, due to the lack of robust evidence, the most recent guideline on atrial fibrillation recommends the implantation of appendage occlusion devices as a IIb indication, level of evidence B-NR, in patients with non-valvular AF at high risk for stroke and with contraindications for long-term oral anticoagulation use.^8^

An ongoing randomized trial (ASAP-TOO)^15^ is seeking to demonstrate the effectiveness of the Watchman prosthesis in this clinical condition, but the study is estimated to be completed in 2023.

Apparently, we are reaching a stage of clinical knowledge and experience in optimizing the use of warfarin and direct-acting anticoagulants in patients with non-valvular AF at high risk of stroke and systemic embolization, recognizing and establishing the safe limits for their use. This opens up a new phase for the consideration of LAA occlusion devices. Therefore, additional prospective, multicenter, controlled clinical trials are needed to clarify the effectiveness and safety of the implantation of the devices in these new clinical situations, such as patients with absolute contraindication to OACs and antiplatelet use, even for a short period of time; patients that had a stroke while receiving apparently effective oral anticoagulation; LAA occlusion as an alternative to chronic use of NOACs; occlusion device implantation simultaneously with AF ablation; in addition to establishing the need and safe handling of short-term anticoagulant therapy and minimal antiplatelet therapy, which should be maintained after the implantation of different prostheses.
